# Electrochemical Aptasensors: Current Status and Future Perspectives

**DOI:** 10.3390/diagnostics11010104

**Published:** 2021-01-11

**Authors:** Abd-Elgawad Radi, Maha Ragaa Abd-Ellatief

**Affiliations:** Department of Chemistry, Faculty of Science, Damietta University, Damietta 34517, Egypt; maharagaa2016@yahoo.com

**Keywords:** electrochemical, aptasensor, label-free, clinically important biomarkers

## Abstract

This article reviews the progress of diversity of electrochemical aptasensor for target analytes detection. The immobilization strategies of aptamers on an electrode surface are addressed. The aptasensors are also introduced in compliance with the assay platforms. Many electrochemical aptasensors are nearly identical to conventional immunochemical approaches, sandwich and competition assays using electroactive signaling moieties. Others are “signal-on” and “sign-off” aptasensors credited to the target binding-induced conformational change of aptamers. Label-free aptasensors are also highlighted. Furthermore, the aptasensors applied for clinically important biomarkers are emphasized.

## 1. Introduction

**Scheme 1:**
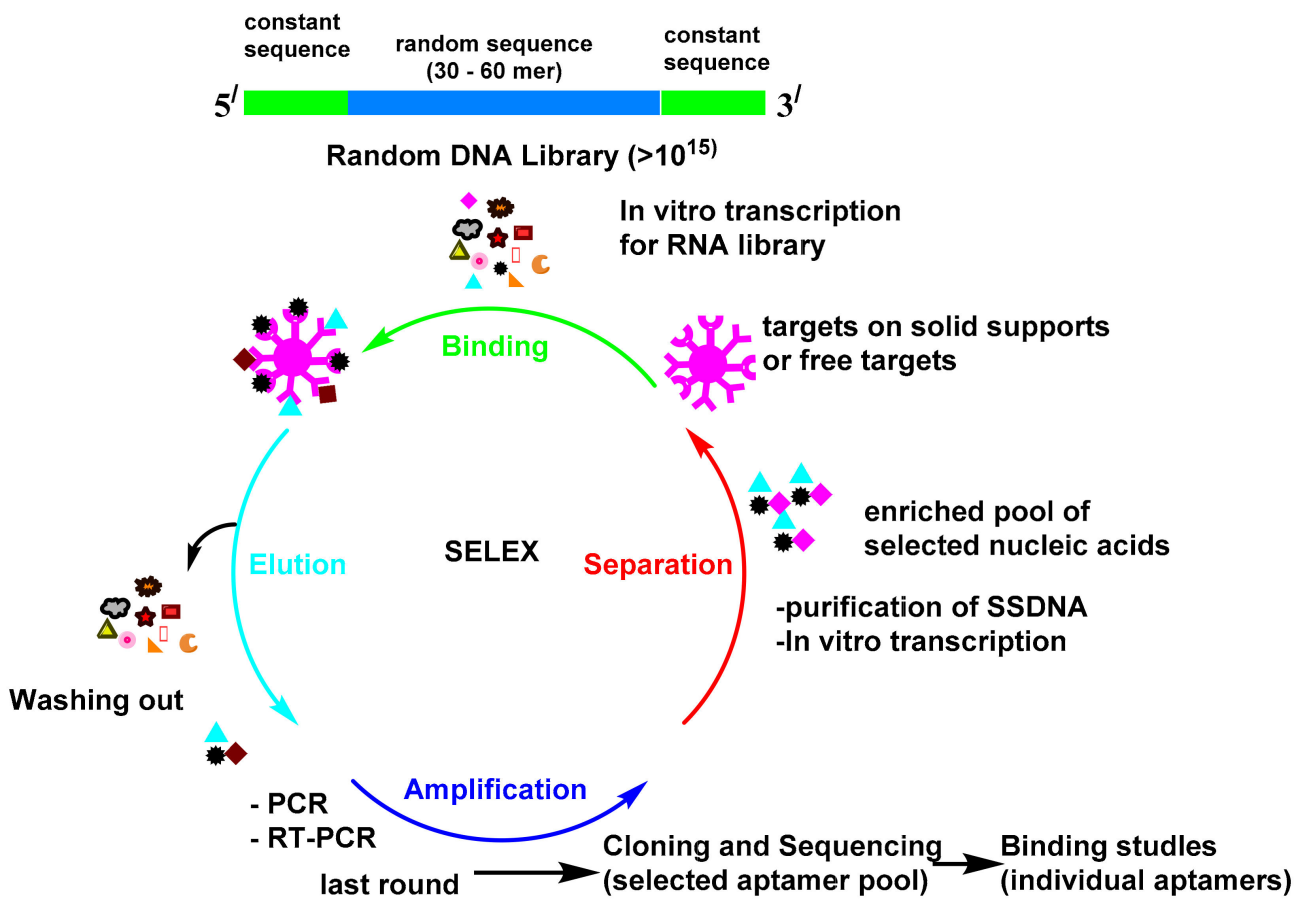
Systematic evolution of ligands by exponential enrichment (SELEX).

The procedure for the in vitro selection of aptamers is called the systematic evolution of ligands by exponential enrichment (SELEX; Scheme 1) [1]. The first reports about SELEX were published by Gold’s and Szostak’s groups in 1990 [2,3]. The SELEX launches with the creation of a multiple library of DNA or RNA molecules. The library is subsequently imported into a target ligand and the sequences, which reveal affinity towards the target molecule and are isolated from the unbound sequences. The bound sequences are then collected and subjected to PCR amplification for the following rounds of enrichment. Multiple rounds have been attained until the library reach to set of convergent sequences with affinity for the target molecule. Since aptamers were found in the 1990s, they have attracted substantial attention and have been utilized in many fields [4]. The antibody-based platform can detect many targets, but it is always hampered by the antibody preparation time, antibody equilibrium, and the effect of the modification on the antibody. The monoclonal antibodies production is very expensive processes [5]. Moreover, immunoassays must be validated in every new batch. Seeking alternatives to antibodies is of enormous importance. Antibodies as proteins are easily denatured and shed their tertiary structure at high temperatures; however, oligonucleotides are more thermally stable and keep their structures over repeated cycles of denaturation/renaturation [6,7]. The aptamer modification by different chemical reactions improve their stability and nuclease resistance [8,9]. The aptamers may be directly chosen in vitro against different targets, from small molecules to proteins and even cells [10]. These advantages make it feasible for them to be used as a recognition probe to substitute antibodies in the diagnostic field. Great attention has been paid to these biosensors, due to their simple utility, accessibility, and real-time outputs. Therefore, sensors that use aptamers as their bio-recognition elements have found applications in diverse fields [11].

A biosensor that is structured from aptamers as a recognition element is called an aptasensor. The electrochemical aptasensor was introduced for the first time in 2004 [12]. Up to now, the best-investigated aptamers are those for thrombin [13,14,15,16,17]. A promising scope for the new era of diagnostics could be offered by the electrochemical biosensors. The principle of function depends on the change in the current, impedance, or potential, in response to the recognition event at the transducer of the sensor [18,19,20,21,22]. The electrochemical detection advantages are low-cost, fast, multi-analyte analyses, readily amenable for miniaturization, as well as being robust and compatible with novel microfabrication technologies and real time detection. These advantages offer the desired characteristics for point-of-care tests for monitoring of clinical analytes.

## 2. Immobilization Chemistry of Aptamer onto the Electrode Surface

The immobilization strategies require the strong linkage of the recognition element to the electrode transducer. The aptamer is frequently tagged with a thiol group, which binds to gold surfaces through Au–S bonds [23]. Mercaptohexanol (MCH) is then added to displace the nonspecifically adsorbed portions of the aptamers and guarantees their vertical orientation, due to the repulsion between the negative alcohol terminus and the negatively charged DNA backbones [24]. The MCH could be subsequently introduced or co-immobilized simultaneously [25]. The DNA loading should be as large as possible for sensitive detection, while at the same time as low as necessary to allow correct folding and avoid steric hindrance. The linker length between the aptamer and its thiol tag must be tuned to enable optimal folding and binding. As a general rule, the longer the alkyl chain, the greater the antifouling properties, but also the more insulating the alkanothiol layer [26,27]. This contributes to a thinner and compact coating layer. The thiol tag is generally introduced at the 5′ end via a C5 linker, to allow sufficient space for aptamer folding and target binding. [28].

Co-immobilization is used as a standard technique. However, the use of MCH has several drawbacks that can lead to the non-reproducibility of manufacture, decreased target binding, and nonspecific binding of interfering molecules that hampers the direct contact with the target analyte and leads to false responses. Self-assembled monolayers are far from ideal and have various types of defects. MCH forms less orderly and more labile monolayers [29], with significant heterogeneity, multilayers, and pinholes, due to the short length of its alkyl chain. In addition, filling with MCH caused a lateral diffusion of previously immobilized aptamers forming DNA islands. The defects of the monolayer negatively influence the conformations of the immobilized aptamers and thus impede the target binding [30].

Alkanethiols cannot completely inhibit electrode fouling, and some fouling agents such as proteins, phenols, amino acids, neurotransmitters, and others, could significantly affect the analytical performance of the biosensor—e.g., sensitivity, reproducibility, stability, and complete reliability—hence, appropriate antifouling approaches will be needed to minimize fouling effect [31].

The aptamer is generally immobilized by a covalent bond between the aptamer and the electrode surface. The reaction of carboxylic acids and primary amines to form covalent amide bonds can be used in the fabrication of aptasensor [32]. One of the most frequently encountered reaction strategies for the aptamer immobilization is the covalent bonding between the aptamer and the electrode surface via a carbodiimide-mediated reaction. The immobilization methods based on diazonium functionalized surfaces by means of electrochemical grafts have been widely used [33,34,35,36,37,38,39], to obtain stable functionalized conductive surfaces for the immobilization of biomolecules.

**Scheme 2:**
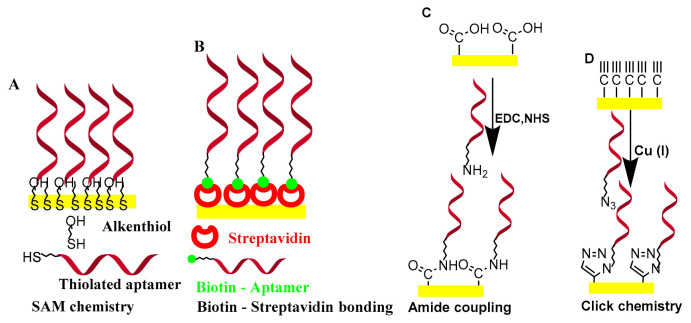
The common surface immobilization methods for aptamers. (**A**) SAM chemistry, (**B**) biotin-streptavidin bonding, (**C**) amide coupling and (**D**) click chemistry.

Click chemistry with an alkyne–azide cycloaddition reaction offers a strong and attractive alternative to traditional chemistry due to its quick and easy purification, biocompatibility, regiospecificity, reactivity and high productivity [40]. The electrode surface is covalently grafted with propargyl acetic acid. The propargyl groups on the electrode can be attached to azide-terminated aptamers by Cu (I)-based click chemistry [41]. As a platform for the immobilization of the aptamer, 10-azidoundecan-1-thiol was self-assembled on an Au surface, followed by the covalent attachment of an alkynyl functionalized aptamer [42]. In the same way, the aptamers were immobilized onto organized mixed layers of diazonium salts using click chemistry. The electrode surface was first modified by electro-grafting of a protected layer of 4-((trimethylsilyl) ethinyl) benzene followed by a second layer of p-nitrobenzene by electrochemical reduction of its corresponding diazonium salts. After deprotection, active ethinyl groups were obtained which reacted effectively with an aptamer, having an azide function in the presence of a copper (I) catalyst, forming a 1,2,3-triazole covalent bond [43]. Copper-free click chemistry on the surface of the acid-functional graphene oxide was also mentioned [44]. The electrically addressable covalent immobilization of probes using click chemistry was used for the simultaneous detection of multiple targets [45]. The common surface immobilization methods for aptamers are shown in Scheme 2.

The signal generated is generally dependent on the probe loading. Gold nanoparticles (AuNPs) are widely utilized to increase the active surface area of the electrode, allowing high loading of immobilized aptamers, and increasing the sensitivity of the sensor. Another interesting approach is the immobilization of aptamers onto new materials, such as gold films/particles, silicates and silicon oxide surfaces, quantum dots of carbon nanotubes and carbohydrates or dendrimers [46]. The dendrimers have great stability and relatively large surface area compared to the flat electrode [47,48], using glutaraldehyde for the crosslinking of avidin on a dendritic surface, hence the immobilization of biotinylated aptamers. Magnetic bead-based assays are of interest and widely used in clinical laboratories due to their compatibility with automation/microfluidics. Multi-walled carbon nanotubes (MWCNT) have been used as modifiers to screen-printed carbon electrodes to immobilize the 5′amino aptamer sequence which has better characteristics than bare SPCE [14].

## 3. Electrochemical Aptasensors Configuration

The direct apta-assay formats are concerned with the molecular recognition of the target using the cognate aptamer immobilized onto the sensing interface. For example, a thiol terminated aptamer was immobilized on an Au electrode. After the thrombin (Tb) binding event, the Tb was detected by evaluation of the thrombin catalyzed reaction that produces p-nitroaniline [49]. The main disadvantage of such an approach is the high detection limits and the limited selectivity of the sensing layer. The biosensing assays could be improved using other different configurations; including sandwich-type, displacement-type and folding-based electrochemical aptasensors. This section discusses advances in the electrochemical aptasensors for target molecules monitoring, focusing on effective detection strategies for the formation of sandwich structures, and EIS measurement of the change of charge transfer resistance, which is related to the target molecule level, and the aptamer conformation change causing the electrochemical signal on/off of the electroactive markers.

### 3.1. Sandwich-Type Electrochemical Aptasensors

The sandwich-type construct involves antibody–aptamer sandwich sensing layers; the antibody is used as a capture element and aptamer as a reporter probe or vice versa. The signaling antibody is usually conjugated with a redox label, such as methylene blue (MB), ferrocene (Fc) or horseradish peroxidase (HRP) which can be detected using electrochemical techniques. [50,51].

**Scheme 3:**
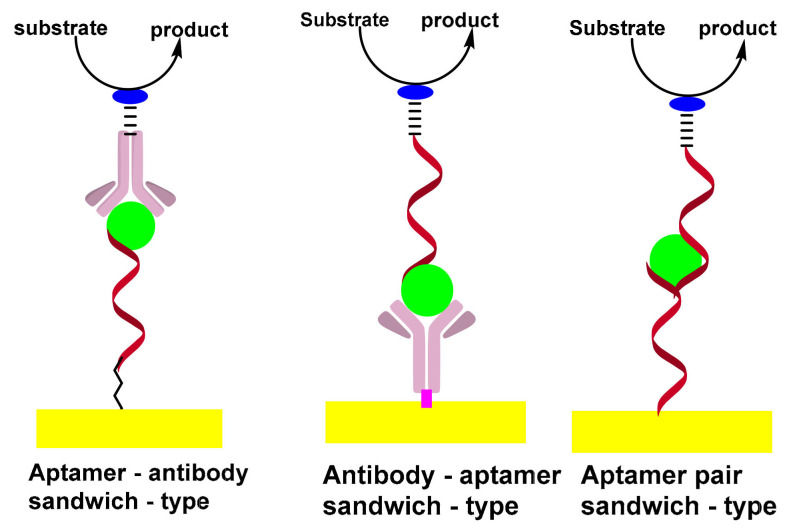
Formation of sandwich-types structures of aptasensing assay.

The sandwich format generally offers higher selectivity as compared to single-receptor approaches, due to the involvement of a pair of receptors; the capture and reporter. In addition, the sandwich format promotes a wide variety of amplification strategies, which can be divided into two broad categories: depending on the nature of synthetic receptors or on the unique advantages of nanoparticles. In addition to DNA amplification, strategies based on the extension of the reporter aptamer with a proper primer thus provoke isothermal and linear RCA [52,53] nucleic acid nanomachines such as DNA walkers [54]. Scheme 3 shows the formation of sandwich-types structures for aptasensing assay.

### 3.2. Displacement-Type Electrochemical Aptasensors

**Scheme 4:**
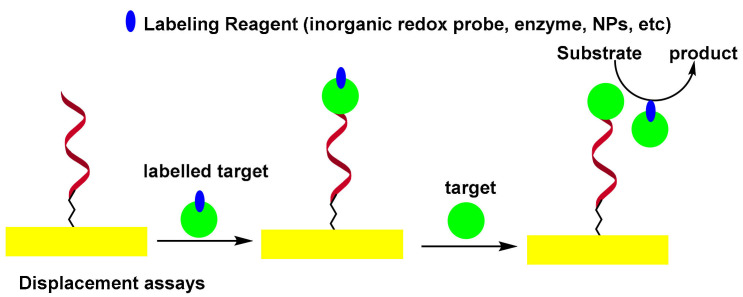
Displacement-type electrochemical aptasensor.

In the displacement assay, the authentic target molecules compete with labeled target molecules for specific binding sites on the sensor surface. It was observed that labeled target molecules used as biocomponents in the displacement test exhibited less affinity than authentic targets towards the binding sites of aptamers [55]. This phenomenon was exploited by labeling the thrombin by peroxidase, thus modifying its structure and allowing it to be replaced by unmodified thrombin. In this format, the peroxidase-labeled thrombin was first captured by self-assembled monolayers (SAMs) of thiolated aptamer on the surface of the Au electrode. The sensor was then incubated with thrombin. The HRP confined to the electrode surface, was measured electrochemically by a H_2_O_2_ peroxidase catalyzed reaction [56]. Scheme 4 shows the displacement-type electrochemical aptasensor.

### 3.3. Folding-Based Electrochemical Aptasensor

For decades, biomolecular recognition has prompted tremendous efforts to adapt biomolecules to the universal platform of molecular sensing. However, biosensors are almost completely unable to play their potential as a reagent-free real-time analysis device. The fundamental obstacle to the wider success of biosensors is that most biomolecules cannot generate easily measurable signals after binding to the target. For example, the antibody will not change its shape when it binds to the recognition target. Therefore, it has proven difficult to convert biomolecule binding events into measurable output signals, especially signals that are generated by the binding of many potentially interfering species in typical biological samples.

**Scheme 5:**
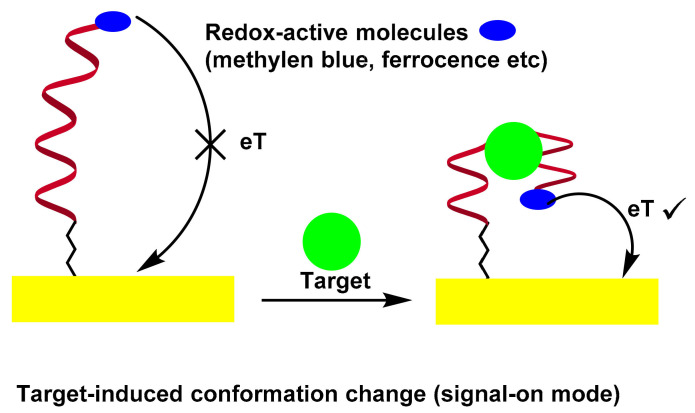
Folding-Based Electrochemical Aptasensor.

A potential solution to solve the problem is based on the binding of electrode-bound DNA probes to induce folding. In this sense, a series of electrochemical nucleic acid sensors using target-sensitive DNA structures with appropriate redox markers can monitor aptamer conformational changes by measuring electrochemical signals, which are directly dependent on the distance between the redox label and the electrode surface [57,58,59,60]. In an early work by Radi et al., a “Switch-on” aptasensor for the detection of thrombin was designed [57]. A thrombin binding aptamer with redox active Fc and thiol at the chain ends was self-assembled on the Au electrode by the S–Au bond. After thrombin interaction, the Fc-labeled aptamer changed its structure from random coil configuration of the aptamer to quadruplex, bringing the Fc label close to the electrode surface to facilitate the electron transfer between the Fc and the surface, as evidenced by the CV and EIS measurements. Scheme 5 illustrates the folding-based electrochemical aptasensor.

Another electrochemical “Switch-off” aptasensor for the detection of thrombin was designed by Plaxco et al. [58]. A 32-base thrombin aptamer modified with thiol and MB at both ends was self-assembled on an Au electrode surface. When Tb was combined with the MB labeled aptamer, the MB marker was distant from the electrode surface and directly led to the weakening of the electrochemical signal.

## 4. Principles of Electrochemical Transduction Techniques

One of the biosensor components is the transducer, which converts the recognition event due to the interaction of the analyte with the recognition element into a measurable sign. The most important electroanalytical techniques are potentiometry, coulometry, voltammetry, amperometry, and electrochemical impedance spectroscopy (EIS). The electrochemical transducer is related to the reaction that involves electrons transfer. Therefore, electrochemical measurements can only be achieved with redox labels that can be attached to the target, recognition element, or soluble redox mediators, which diffuse onto the electrode surface to be reduced or oxidized by heterogeneous electron transfer. The redox probe includes redox enzymes, inorganic catalysts, or nanoparticles. The diffusion kinetics of the redox mediator to the electrode is influenced by the surface state of the transducer. Thus, the binding of the analyte affects the diffusion efficiency, electron transfer, and finally the detected signal. Other transduction approaches are connected to the intrinsic electroactivity of guanine-rich guanine aptamer on a graphite pencil electrode [61].

Nanoparticles can charge several units of electroactive species (ferrocene or thionine (Th)), and are thus used for signal amplification [62]. An efficient strategy is to load one or more enzymes (natural peroxidase and G-quadruplex/hemin (GQH) DNAzyme) as biocatalysts onto nanomaterials to improve the sensors’ sensitivity through enzymatic electrochemical processes; for example, a signal enhancement has been achieved by PCN-224-Pt nanoelectrocatalysts, natural HRP, and GQH HRP-mimicking DNAzymes catalyzing the oxidation of HQ with H_2_O_2_ [63]. Natural redox enzymes could be replaced by catalytic nanomaterials as reporter molecules. In this respect, the nanoparticles CuO [64] and Pd–Au [65] show hydrogen peroxide reduction activity. Although they have the best stability against temperature and pH changes, their peroxidase activity is lower than that of the natural counterpart.

## 5. Electrochemical Aptasensors for Clinical Application

### 5.1. Electrochemical Aptasensors for Cardiac Biomarkers

Cardiovascular disease (CVD) is one of the most common causes of death. Urgent treatment of patients with chest pain is crucial. The practical tests are the measurement of aspartate aminotransferase (AST), lactate dehydrogenase (LDH), creatinine kinase (CK-MB) and troponin, which are the main biomarkers of myocardial infarction (MI). Therefore, a portable, fast, and inexpensive biosensor device for the detection of cardiovascular diseases is a highly necessary element to monitor the onset and progression of cardiovascular disease quickly and accurately [66]. Troponin contains a combination of three protein subunits (troponin T (Tnt, ~35 kDa), troponin I (Tni, ~30 kDa) and troponin C (Tnc, ~18 kDa)) [67,68]. The increased troponin levels in patients, along with other clinical features, are the best tool for the rapid diagnosis of MI. Other important diagnostic biomarkers of MI are myoglobin (Mb) [4,5,6,69,70,71] and brain natriuretic peptide (BNP) [72,73,74] and so on.

An electrochemical aptasensor for cardiac troponin I (cTnI) was designed [75]. The FC-modified silica nanoparticles Fc-SiNPs were synthesized for use as a redox probe and the cTnI -aptamer was immobilized on the surface of the Au electrode. The self-assembled aptamer monolayer allows easy access of Fc-SiNPs to the electrode surface, resulting in an enormous current response. On the other hand, the specific interaction between cTnI and aptamer prevents the approach of Fc-SiNPs, leading to considerable decrease in the peak oxidation current of SWV.

The aptamer-based electrochemical detection of cTnI was also described [76]. The gold nanoparticles AuNPs were electrodeposited onto Ti foil. Then, the SH-functionalized DNA aptamer was immobilized onto the AuNPs/Ti electrode surface [77]. A similar aptasensor is composed of an array of Au nanodumbbells, synthesized using putrescisin as a form directing agent at the surface [78] to immobilize TnI 76-mer on the transducer to detect TnI. The DPVs in the presence of ferro/ferricynaide anions was used to monitor the signal. The ferro/ferricynaide anions have a certain proximity to the aptamer surface and generate a certain peak current, due to the net negative charge of the phosphate backbone. On the other hand, TnI also has a net negative charge and the combination of TnI and the aptamer resulted in an increase in the negative surface charge of the aptamer, repelling ferro/ferricynaide anions from the sensor surface. In this way, both aptasensors act as a signal-off device [77,78].

An SPCE-based sandwich aptamer for the detection of cTnI has been designed [79]. The SPCE was functionalized using AuNPs that were electrically deposited onto the SPCE, and a 2,2′:5′,2″-terthiophen-3-carboxylic acid monomer (TTCA) was electrically polymerized to provide a modified carboxylic surface. Following activation by the EDC/NHS, the amine-modified aptamer (Tro4) was covalently immobilized on the SPCE to act as a capture probe. After binding of cTnI to the aptamer (Tro4), the hydrazine modified aptamer (Tro6) as a capture probe was later incubated with the electrode, and the amperometric signals were recorded in H_2_O_2_ solution. The aptasensors produced exhibited high analytical performance with a detection limit well below existing cut-off values.

A troponin I aptasensor (GC/N-prGO-aptamer) was manufactured. The initial stages involved the syntheses of O-(2-aminoethyl) polyethylene glycol modified pyrene (py-PEG12-OH) Py-PEG, the reduced graphene oxide (rGO) from the GO precursor using hydrazine reduction, and the porous doped N reduced graphene oxide (N-prGO). The GCE has been modified by drop casting to provide the GC/N-prGO electrode. The GC/N-prGO electrode was further modified by 1-pyrenecarboxylic acid and pyrene-PEG. The aptamer Tro4 was immobilized by activating the carboxyl groups by EDC/NHS. In the final stage, a troponin I aptasensor (GC/N-prGO-aptamer) was manufactured for the detection of cTnI, by DPV of [Fe(CN)_6_]^4−^ [80]. The aptamer-MoS_2_ nanoconjugates was proposed as a sensing platform for the detection of cTnI [81]. For comparison, Au@Sio2 AuNPs were also used to detect cTnI by EIS.

A sandwich-type aptamer-based nanoprobe is created from GQH DNAzymes/dAPT/HRP/AU@PtNP/Fe_3_O_4_@UiO-66 and AU@PtNP, assembled on Fe_3_O_4_@UiO-66 via free amino groups, followed by thiol-modified aptamers (SH-2G-Tro4 and SH-2G-2G-2G-TH-2G) integrated with the double G-G sequence quadruplex, anchored to Au@PtNPs by thiol–metal interactions [82]. The HRP was then decorated with AU@PtNP, and the DNAzyme GQH mimicking catalytic HRP was assembled in the presence of hemin and potassium ions. Next, the dAPT-bound thiol NTH DNA (Tro4 and Tro6) was immobilized on the Au surface by Au–S interactions to capture cTnI. The GQH DNAzyme/dAPT/HRP/Au@PtNP/Fe_3_O_4_@UiO-66 nanoprobe has a high affinity for the recognition of cTnI and amplification of the current signal by catalyzing the oxidation of hydroquinone (HQ) to benzoquinone (BQ) with H_2_O_2_.

An approach based on the specific recognition between cTnI and the aptamer of cTnI and terminal deoxynucleotidyl transferase (TdT)-mediated signal amplification was described. [83]. The Au electrode was modified with probe 2 (P2), in the presence of cTnI, the aptamer of cTnI that in probe 1 (P1)/aptamer complexes bond with cTnI specifically and release the free P1. P1 would bind with P2, resulting in the formation of 3′-OH of DNA. In the presence of terminal deoxynucleotidyl transferase (TdT) and dTTP, TdT mediated P1 to extend and formed the structure of poly T. Thereafter, MB-poly A hybridized with the extended poly T and generated an electrochemical signal. This sensor was also successfully applied to the detection of cTnI in numerous spiked biological samples.

A label-free electrochemical detection of Mb was also realized using aptamer functionalized rGO/CNT nanostructured electrodes by measuring its direct electron transfer [84]. For the detection of Mb, an electrochemical aptasensor has been formulated from the Y-shaped structure of the aptameric double conjugated wire (DApt), aptamer (CS), Au electrode, and the exoonuclear I (Exo I) [85]. In the absence of Mb, the Y-shaped structure remains intact and a weak electrochemical signal is observed. After the target addition, the DApt detaches from the CS and connects to the Mb, causing the Y structure to be removed, and an enhanced signal can be recorded after the addition of Exo I. The electrochemical determination of Mb was demonstrated by measuring the direct electron transfer of the redox active Mb on an aptamer-functionalized black phosphorus nanostructured electrode [86]. Poly-l-lysine (PLL) has been used to functionalize the black phosphorus nanosheets to enhance the binding with the anti-Mb DNA. An electrochemical aptasensor has been prepared from graphene-conjugated AuNPs (TCPP-Gr/AuNPs) and meso-tetra (4-carboxyphenyl) functionalized porphyrin which provide a large surface area to effectively immobilize the myoglobin binding aptamer (Mb-BA) for Mb detection [87]. A label-free aptasensor was successfully built by grafting Mb-binding-aptamer (MBA) onto AuNPs/arginine-glycine-aspartic/carboxylated graphene/GCE (Au/RGD/GR-COOH/GCE) surface [88] and was employed for Mb detection by DPV. The Mb-aptamer, immobilized on the surface of a composite of PtSn nanoparticles and carbon nanotubes (PtSnNP/CNTs) on GCE, was used for the electrochemical detection of Mb [89]. The amperometric detection was based on the suppression of the electron transfer of the hexcyanoferrate used as a redox probe which was induced by the conformational change of Mb-aptamer in the presence of Mb. A boron nitride nanosheets (BNNSs) decorated with gold nanoparticles (AuNPs/BNNSs) were used to construct an aptasensor for the detection of Mb [90]. The BNNSs were deposited on the fluorinated tin oxide (FTO) electrode, and the AuNPs were then deposited on the BNNS/FTO electrode. The thiol-functionalized aptamer (Apt) was immobilized by Au–S covalent bond with the AuNPs/BNNSs/FTO electrode. The redox [Fe(CN)_6_]^3/4−^ probe was used to observe the change in oxidation current, while Mb target was bound to the sensor surface.

Impedimetric aptasensing for Mb and cTnI has utilized nanodiamond heteronostructure (NDs) and hydrogen-substituted graphidine (HsGDY) [91]. The HsGDY@NDs nanostructure can immobilize several chains of aptamers to detect the target biomarkers. An aptamer with a unique affinity was used to build a label-free electrochemical aptasensor super-sandwich for the detection of Mb, based on target-induced aptamer displacement.

Multi-analyte detection of cardiac biomarkers, cerebral natriuretic peptide (BNP-32), and cardiac troponin I (cTnI) was performed using electrochemical aptasensors [41]. The Au electrode was modified with polyethyleneimine/reduced graphene oxide films. The covalent grafting of propargylacetic acid incorporated propargyl groups on the electrode to which the azide-terminated aptamers can be immobilized using Cu (I)-based click chemistry. The modification of aptasensors with pyrene anchors carrying poly (ethylene glycol) units avoided the biofouling.

The properties of different cardiac biomarkers aptasensors are displayed in Table 1.

### 5.2. Electrochemical Aptasensors for Alzheimer’s Disease (AD) Biomarkers

Alzheimer’s disease (AD) is the most common neurodegenerative disorder and remains incurable. Reliable measurements of AD biomarkers such as tau protein, β-amyloid protein (Aβ) and apolipoprotein E4 may speed up the early diagnosis of AD. Early diagnosis and intervention are crucial to delay the onset and progression of the disease. The AD is often associated with a low level of adenosine triphosphate (ATP). Therefore, the detection of ATP is also a credible AD biomarker.

The detection of Tau381 is carried out using a label-free electrochemical aptasensor. The aptamer has been immobilized on a graphene/thionine/gold nanoparticles/glassy carbon electrode [92]. The thionine, conjugated the carboxylic graphene with the gold nanoparticles, generated the electrical signal. The tau antibody (anti-tau) and an aptamer specific to tau-381 were used to build a sandwich-type sensor for the detection of tau-381 [93]. The cysteamine-stabilized AuNPs was utilized for the amplification of DPV signals recorded in response to the tau-381. Similarly, the detection of the oligomers (AβO) was performed by an antibody–aptamer sandwich assay [94]. The antibodies of the AβO and aptamer were used as recognition elements and detection probes, respectively. The signal amplification by high loading of Th on the AuNPs ensures a low limit of detection. An aptasensor has been developed for screening of the Aβ [95]. The RNA aptamer was immobilized on a gold leaf nanostructure. The Aβ bonding with the aptamer was monitored by the ferro/ferricyanide redox probe. An ssDNA aptamer, receptor specific to AβO, was also self-assembled to the Au rod electrodes through Au–S interactions to construct EIS-based aptasensors for AβO assay [96]. A sandwich-type aptasensor based on metal–organic structures (MOFs) as signal probes has been developed for the detection of AβO [97]. A modified aptamer-attached gold nanoflower (AuNF) electrode has been used for the capture of targets and aptamer-labeled gold nanoparticles/cu-MOF conjugates (AuNPs/cu-MOF) for the generation of signal.

The detection of α-syn, an oligomeric biomarker linked to Parkinson’s disease and AD, has been described [98]. The complementary aptameric band (CS) has been directly immobilized at the Apt site on the electrode surface to prevent the self-accumulation of a single-chip Apt strip complex and retains the Apt activity. In the absence of the α-syn oligomer, TdT expands the length of Apt and CS, thereby enhancing the accumulation of MB as a redox agent on the electrode surface resulting in a high current signal.

A multi-electrode array (MEA) of Au microelectrodes modified with 3D electrodeposited nanostructures (3D-GMEs) was used to immobilize the thiol and Fc-modified stem-loop oligonucleotide aptamers receptors specific to AβO or ATP [99]. The aptamer stem loop receptor for AβO has a closed structure, with the Fc closely approaching the electrode surface, facilitating electron transfer, and causing a high blank current signal. After attaching the AβO target to the aptamer receptors, the stem loops were changed and pulled the end of the Fc-labeled DNA away from the surface, thereby blocking electron transfer and resulting in the signal-off. Afterwards, a predefined set of microelectrodes was regenerated by electrochemical desorption to allow the attachment of a second aptamer receptor for ATP. The binding of the ATP to the sensor caused an increase in signal, corresponding to an activated current signal due to a conformational change, which caused the aptamer with its ferrocene redox group to bend closer to the electrode surface.

The specifications and electrochemical performance of aptasensors for AD biomarkers are collected in Table 2.

### 5.3. Electrochemical Aptasensors for Infectious Disease Biomarkers

The spread of infectious diseases around the world threatens humanity. Early detection of infectious pathogens or their markers is essential for clinical diagnosis. Detection methods for infectious pathogens are time-consuming and require expensive equipment and qualified personnel. Aptamer-based technologies are promising diagnostic tools that combine rapid pathogen detection, simplicity, ease of use, operational efficiency, accuracy, and cost-effectiveness with the trend towards portable platforms suitable for field testing [99,100].

The release of interferon (IFN)-γ by T-cells in the blood is used for the diagnosis of tuberculosis (TB). A DNA aptasensor specific for IFN-γ is formed from an A hairpin containing an IFN-γ binding aptamer which has been thiolated, conjugated to methylene blue (MB), and immobilized on an Au electrode [101].

The IFN-γ bonding triggered the aptamer hairpin to unfold, displacing the redox MB molecules away from the electrode and lowering the electron transfer efficacy as measured by SWV. The bovine gamma interferon (BoIFN-γ) was also assayed [102]. A graphene-based FET monolayer structure is integrated on a PDMS substrate with an IFN-γ aptamer attached to the graphene [103]. A charge distribution in the electrolyte has been changed by the target, resulting in an increase in electron transfer efficiency. The silicon nanowires SiNWs were coated with gold and were further functionalized with thiolated aptamers specific for IFN-γ to improve the sensitivity of the IFN-γ detection, [104].

A microsystem of several Au electrodes for the simultaneous determination of two cytokines, IFN-γ and TNF-α, was constituted [105]. The aptamers, specific to either IFN-γ or TNF-α, were thiolated for assembly on Au electrodes and functionalized with an MB redox reporter for signal transduction. An adsorption/desorption cycle was used to assemble MB-functionalized aptamers onto individually addressable electrodes. The secreted IFN-γ and TNF-α were monitored by performing SWV at individually addressable electrodes. This platform was applied to monitor the rate of cytokine release from primary T cells and monocyte cell lines.

Clostridium perfringens is a common pathogen that is easily viable and escapes detection. A molecular beacon was formed from the labeled graphene/GCE and the nanocomposite ADH/hemin/G-quadruplex/Fe_3_O_4_ [106]. The beacon forms a hairpin and obstructs the binding of the aptameric streptavidin (SA), whereas the target DNA opens the hairpin and exposes the aptamer region of the streptavidin (SA) to capture the nanocomposite SA/ADH/hedin/G-quadruplex/Fe_3_O_4_. SA/ADH/hedin/G-quadruplex serves not only as HRP imitating DNAzyme, but also as NADH-oxidase and NADH-peroxidase imitating DNAzyme. Therefore, in the presence of H_2_O_2_ and NADH, hemine/G-quadruplex and DHA effectively catalyze the conversion of alcohol to acetaldehyde and enhancing the DPV signal.

An aptasensor based on the specific interaction between AuNPs, polyethyleneimine (PEI), aptamer NS1 and the NS1 protein, has been proposed for the detection of the NS1 protein of the dengue virus [107]. The well-dispersed AuNPs attached to the surface of the electrode repel the [Fe(CN)_6_]^−3/−4^ anions from the interface, thus reducing the current signal as compared to the bare electrode. The cationic PEI caused the aggregation of the AuNPs, opening up access to the electrode surface, and increasing electron transfer. The NS1-aptamer has formed an aptamer/polymer duplex, reducing the current signal. The analyte (NS1) is freed from the aptamer as an aptamer–aptamer duplex and forms a more stable aptamer/target complex, followed by a significantly higher current signal.

The specifications and electrochemical performance of aptasensors for infectious disease biomarkers are compiled in Table 3.

### 5.4. Electrochemical Aptasensors for Cancer Diagnosis

Tumors are caused by the infinite proliferation of normal cells in a living body and are a serious threat to human health. A tumor marker is a class of substances produced by the development of tumor cells that release during tumor proliferation. Polymerase chain reaction (PCR) and immunohistochemistry techniques are commonly used to detect tumor biomarkers, but are generally laborious and time-consuming. The electrochemical method has the advantages of simplicity, low cost, and high sensitivity. Aptamer-based biosensors have been successfully developed to detect tumor biomarkers using outstanding aptamer characteristics such as appropriate synthesis and controlled modification, high binding affinity and specificity, good stability, low toxicity, rapid tissue penetration and low batch-to-batch variability; enhancing detection sensitivity by applying amplification [108].

#### 5.4.1. Electrochemical Aptasensors for Circulating Tumor Cells (CTCs)

CTCs have been revealed to be an important key to tumor metastasis; therefore, their detection plays a fundamental role in the diagnosis and prediction of cancer [109].

An aptasensor was made from two cell specific aptamers, TLS1c and TLS11a, to recognize MEAR cancer cells [110]. Both aptamers are attached to the GCE surface via linkers; TLS1c via a flexible linker and TLS11a via a rigid linker. The double-modified GCEs ss-TLS1c/ds-TLS11a allow better recognition than those modified with only one type of aptamer alone or ds-TLS1c/ds-TLS11a with both rigid linkers.

An impedimetric cytosensor for CTCs detection was outlined [111]. The cytosensor has specific recognition for epithelial cell adhesion molecules over-expressed on the cell membrane and the EpCAM aptamer. MCH interspaces on the Au electrode were filled by capture probe. The CTCs in a real blood sample can be easily distinguished by the cytosensor. In addition, the cytosensor can be reused by cleaving the deoxyuridines (dUs) of the aptamer.

A nanochannel–ion channel hybrid structure for CTC detection has been established [112]. The PAA membrane was clamped between two plastic films of poly (dimethylsiloxane) (PDMS) and placed between two-half of electrolytic cell. The aptamer sgc8c probe was then immobilized on the lateral surface of the ion channel, which can strongly bind to the transmembrane receptor protein tyrosine kinase 7 expressed on the membrane of CCRF–CEM cells. Thus, the trapped CCRF–CEM cells block the ion flow through the ion nanochannel. The CCRF–CEM cells are liberated from the AAP by benzonase nuclease, resulting in the recovery of the ion flow through the nanochannel–ion channel hybrid. The variation of the ion flow can be monitored by the LSV technique, enabling the label-free detection of CTCs.

An efficient method to detect human leukemic lymphoblasts (CCRF–CEM) using aptamer-modified Au nanowire arrays (AuNWs) has been reported [113]. The AuNWs were electrodeposited using anodic aluminum oxide (AAO) as the template. The AuNW-aptamers have higher specificity and capability to capture the target cells. Moreover, the captured CCRF–CEM cells can be freed from AuNWs by an electrochemical desorption process.

An electrochemical cytosensor has been developed to detect CTCs using reduced GrO/AuNPs composites as the support [64]. The MCF-7 CTCs were detected by cytosensor with efficient recognition between the specific mucin 1 protein (MUC-1) over-expressed on MCF-7 cell membranes and the MUC-1 aptamer. The nanozyme CuO served as a signal-amplifying nanoprobe in the cytosensor to detect CTCs.

A multiplexed sensor for the monitoring of CTCs has been demonstrated [62], using the specific aptamer-functionalized AuNPs–Fe_3_O_4_–GS capture probes and the aptamer/electroactive species-loaded AuNP as amplification signal probes. The specific aptamers immobilized on the AuNP array-decorated magnetic graphene sheets enable efficient recognition and capture of the target CTC cells and the electroactive species-loaded AuNPs lead to significant signal amplification, thus results in the successful monitoring of two rare CTCs.

An aptasensing platform was used to quantify CTCs [114]. The MB-modified, rod-loop-shaped aptamers were used as a recognition element for the K562 cells. Thiolated complementary strands hybridized with MB aptamers to form double-stranded DNA (dDNA) which were then self-assembled on the surface of AuNP-modified Au electrode, leading to a marked MB signal. In the presence of K562 cells the Mb-aptamers recognise and bind with the cells, causing the MB-aptamers to disassemble from the GE surface and resulting a signal-off MB signal.

An electrochemical assay for detecting CTCs has been reported [115]. Anti-epithelial cell adhesion molecule (EpCAM) antibody modified with Fe_3_O_4_ magnetic nanospheres (MNs) were utilized to capture breast cancer cell MCF-7. Aptamers can be linked onto LiFePO4/Au via the Au–S bond and then the LiFePO_4_/Au–aptamer complex can effectively capture the MUC1 protein that is over-expressed on the cell surface of MCF-7. The AuNPs modified LiFePO_4_ LiFePO4/Au) composite was chosen to generate the electrochemical signal by the reaction of phosphate in LiFePO_4_ with molybdate to form redox molybdophosphate.

An electrochemical aptasensor based on dual signal amplification by CeO2@Ir nanorods (Ce@IrNRs) and enzyme-free DNA walker, was developed for the simultaneous detection of CTCs [116]. A membrane protein MUC1-targeting aptamer was used to recognize and capture MCF-7 cells. The deoxyuracils of the aptamer were hydrolyzed by uracil DNA glycosylase to isolate the captured cells. The Ce@IrNRs had high peroxidase activity, and the enzyme-free DNA walker released more signal probes combined with Ce@IrNRs, resulting in amplification of the signal.

A direct amplification-free method for the detection of CTCs using MCF-7 as a model has been proposed, using ferroceneboronic acid (FcBA) and 4-mercaptophenylboronic acid (MPBA) as the recognition element and signal probe, respectively [117]. FcBA can be measured by DPV, and the AuNPs aggregation could be triggered by MPBA. The CTCs captured by magnetic bead-modified aptamer (Apt-MBs) can sequester FcBA or MPBA molecules by the formation of boronate ester bonds, leading to a decrease in the electrochemical signal of FcBA by avoiding MPBA-triggered AuNPs aggregation. The overexpression of sugar groups on the CTCs surface made it possible to avoid the use of additional antibodies or aptamers to recognize the captured cells.

The specifications and electrochemical performance of aptasensors for CTCs are gathered in Table 4.

#### 5.4.2. Electrochemical Aptasensors for Carcinoembryonic Antigens (CEAs)

CEAs are generally found at very low levels in the blood of healthy individuals, however levels can be elevated in certain types of cancers such as colon and rectal, pancreatic, breast, ovarian, or lung cancer, allowing CEAs to be used as tumor markers in clinical tests [118]. A simple sandwich electrochemical aptasensor for the detection of CEA has been developed [119]. The thiol-terminated CEA aptamer-1 (Apt1) has been self-assembled on the Au electrode to capture the CEA target, which is capable of binding the capped AuNPs–Apt2 conjugates. The DPV response of Fc was exploited to measure the CEAs.

An electrochemical aptasensor has been manufactured for CEA detection [120]. The CGE with electrodeposited AuNPs was used for the immobilization of CEAapt1 by Au–S affinity. The thiol-terminated CEAapt2 (CEAapt2) and toluidine blue (Tb) with dendritic Pt@AuNWs resulted in the formation of the AuNWs–CEAapt2–Tb bioconjugate. After CEA capture, the bioconjugate completed the sandwich pattern. The response was enhanced due to the favorable catalytic capacity of the dendritic Pt@AuNWs with peroxidase mimetic activity towards H_2_O_2_ reduction, accelerating Tb electron transfer.

A regenerable electrochemical aptasensor was reported for the parallel detection of mucin 1 biomarkers (MUC1) and CEA [121]. The DNA1 was the first self-assembled Au electrode. The sulfhydryl-modified DNA1 as a coupler was designed to be complementary to DNA2 (MUC1 aptamer) and DNA3-MB (CEA aptamer). Therefore, DNA2 and DNA3-MB can be immobilized on the Au electrode by hybridization with DNA (DNA1) to form the detection interface (Au/DNA1/MCH/DNA2/DNA3-MB/Au). The MB was relatively far from the electrode surface and electron transfer was impeded. After MUC1 bonding, the MUC1–aptamer complex was dissociated and transferred to the solution, and the distance between the remaining redox probe and the electrode surface was shortened to facilitate electron transfer. When the target ACE was introduced, the DNA3-MB/ACE complex was separated from the DNA-ds and the MB signal was therefore reduced. Therefore, MUC1 or ACE detection was performed by monitoring the MB signal changes.

A label-free aptasensor for CEA based on ternary nanocomposite of gold, hemin and graphene on the glassy carbon electrode has been envisaged [121]. The aptamer was assembled on the AuNPs on the electrode surface by covalent bonding of Au–S. The hemin absorbed by the graphene nanoparticles acts as an in-situ probe, due to its excellent redox properties. The current changes caused by the CEA specifically attached to the aptamer-modified electrode are exploited as a signal for CEA detection.

A bipolar electrode system (BPE) using Prussian blue (PB) has been proposed for the electrochromic detection of CEA [122]. The CEA aptamer was pre-anchored on the anode pole to capture the CEA through a strong link. The presence of CEA as a high molecular weight protein on the electrode surface significantly hindered the interfacial electron transfer kinetics of the redox probe ([Fe(CN)6]^3−/4−^) and led to lower electrochemical currents which reduced the amount of PB deposited at the BPE cathode. The amount deposited depended on the concentration of CEA and could be observed with the naked eye. This electrochromic platform provides a fast and visual method for the detection of CEAs.

A triple signal amplification strategy was applied to detect CEAs [123]. Aptamer and hairpin-shaped oligonucleotide functional gold nanorods (HO-GNRs) were as used as the recognition element and signal amplifier, respectively. The HRP and HO aptamer with biotin on terminal 3 and thiol on terminal 5′ were carried by the GNR. When the target binds to the looped parts of HO, its loop structure is opened and exposes the biotin to react with the pre-immobilized streptavidin. The HRP/H_2_O_2_/o-Phenylenediamine catalytic reaction cycle triggered a significant electrochemical signal.

An impedimetric aptasensor has been proposed for CEA analysis [124]. Pt Pd nanowires (PtPdNWs) were used as nanocarriers to significantly immobilize CEA aptamers. Next, two DNA hairpins, H1 and H2, were inserted to activate the chain hybridization reaction (HCR) using the DNA initiator, resulting in the formation of a long DNA scaffold. Meanwhile, molecules mimicking the enzyme, manganese (III) meso-tetrakis (4-n-methylpyridiniumyl)-porphyrin were incorporated into the resulting DNA, generating the MnTMPyP-dsDNA complex with peroxidase-like activity. Under biocatalysis of 3,3-diaminobenzidine (DAB) by MnTMPyP-dsDNA a non-conductive IPS is formed, resulting in a significantly amplified impedance signal due to the high barrier to electron transfer between the electrode interface and the redox probe.

An enzymatic polymerisation strategy based on a pair of specific DNA aptamers has been set up for the CEA analysis [125]. One aptamer is thiolated at the 3′ end and immobilized on the Au electrode as a capture probe, while the other has a thiol group at its 5′ end and is modified with AuNPs to form a nanoprobe. The two aptamers can “sandwich” the target, and then the terminal deoxynucleotidyl transferase (TdT) catalyzes the binding of the biotin-labeled dNTPs to the 3′-OH terminals of the DNA aptamer on the nanoprobe, which bind to many avidin-modified horseradish peroxidases (Av-HRP), resulting in a huge amount of HRP-catalyzed reduction of the peroxide.

A hybrid material composed of molybdenum selenide, graphene and gold nanoparticles (MoS_2_/graphene/AuNPs) has been used to construct an aptasensor with high-aptamer loading for the CEA determination. The thiol-labeled CEA-binding aptamer was bound to the electrode through Au–S bonds to recognize the CEA [126].

A label-free impedimetric aptasensor has been proposed for the detection of CEA based on the interaction between CEA and an aptamer immobilized on the surface of a mesoporous silica nanoparticle/gold nanoparticle (AuNPs/AMCM)-modified GCE [127]. The MCM was functionalized by amino groups, and then AuNPs were embedded into the amino-functionalized MCM–41 (AMCM). Using the EIS method, the developed aptasensor was successfully applied for CEA detection.

Aptasensors based on aptamer-embedded zirconium-based metal-organic framework composites (509-MOF@Apt) were developed to detect various target molecules: thrombin, kanamycin, and CEAs [128]. The platform created depends on the conformational changes resulting from the bonding interactions between aptamer strands and targeted molecules.

To prepare an aptasensor for CEA detection, the antibodies against CEA covalently conjugated with the graphene oxide (GO) surface were initially immobilized onto the GCE surface [129]. Antigens (CEAs) and gold nanorods (GNR) modified with the CEA aptamer were collected sequentially on the surface of the GCE in a sandwich format. The molybdate reacted with the phosphate groups of the aptamer backbone to form an electroactive molybdophosphate precipitate on the surface of the GCE, leading to a highly amplified signal. The redox current was ascribed to electron-transfer between different states of Mo in the formed molybdophosphate (PMo_12_O_40_) precipitates.

An integrated signal probe (ISP)/aptasensor based on AuNPs was used for dual analyte detection of Mucin 1 (MUC1) and CEAs [130]. Two different aptamer probes labeled with MB and Fc were used as Signal1 (sP1) and Signal2 (sP2) probes. As a result, the structural switching of DNA induced by the binding of targets to aptamers made it possible to detect MUC1 and CEA individually or simultaneously by monitoring the electrochemical responses of MB and Fc.

An aptasensor based on the zirconium metal–organic framework nanocomposite (ZR-MOF, UiO-66) incorporated with silver nanoclusters (AgNCs) using CEA-targeted aptamers as a template (AgNCs@Apt@UiO-66) has been reported [131]. An aptasensor based on the redox-active molybdophosphate, in combination with a rolling circle amplification (RCA) was also used for the detection of CEA. AuNPs were used as a carrier for the aptamer and primer loading. The sandwich structure of anti-CEA/CEA/AuNP-aptamer-primer on a GCE enabled an efficient RCA reaction. SWV was applied to record the current signal.

An impedimetric aptasensor for the CEA was based on cascade catalysis for signal amplification. First, Cu-based MOF nanocarriers were loaded with Pt nanoparticles (PtNPs), obtaining Pt@CuMOFs with peroxidase-like activity [132]. Next, the G-rich CEA aptamer, hemin, and GOx were immobilized on Pt@CuMOFs. Then, the CEA aptamer was switched to hemin/G-quadruplex (hGq), resulting in Pt@CuMOFs–HGQ–GOx as signal probes. The addition of 3,3-diaminobenzidine (DAB) and glucose catalyzed the oxidation of glucose and generated H_2_O_2_ in situ. Meanwhile, Pt@CuMOFs and hGq with mimicking peroxidase activity catalyzed the decomposition of H_2_O_2_, which, accompanied by the oxidation of DAB and the formation of insoluble non-conducting precipitates, led to a substantially enhanced electrochemical impedimetric signal.

An aptasensor based on graphene quantum dot ionic liquid-Nafion (GQDS-IL-NF) composite film and Pb^2+^ dependent DNAzyme assisted recycling for CEA determination has been described [133]. The heightened sensitivity of the aptasensor was based on the signal amplification protocol. In the presence of CEA, hairpin DNA recognized the target and then formed a CEA–aptamer complex. The DNAzyme sequence hybridized with MB-labeled substrate chain. In the presence of Pb^2+^, the complementary pair of double-stranded DNA was cleaved, the CEA–aptamer complex was freed, and a DNAzyme-assisted signal amplification reaction was conducted, yielding many MB substrates. Next, the GQDs-IL-NF composite film was dispersed on the surface of the CEAs to interact with the MB substrates in a non-covalent π–π stacking interaction, producing a large initial electrochemical signal.

A sandwich-type aptasensor for the detection of CEAs has been constructed using hemine/G-quadruplex conjugated peptides (HGQ-peptide) and porous Pd nanoparticles (PdNPs) [134]. The hGQ-peptide displayed an improved catalytic activity over unmodified HGQ. The porous PdNPs served as a carrier for effective immobilization of HGQ-peptide hybrids, aptamers, redox-active toluidine blue (Tb), and alcohol dehydrogenase (ADH), leading to the generation of D’ADH/TB/HGQ-peptide/aptamer/PdNPs as bioprobes. Upon the specific reaction between the aptamer and CEA, the bioprobe showed a very efficient signal amplification obtained by the cascade enzymatic reaction catalyzed by the bioprobe.

In order to detect CEA, a label-free and lectin-based sandwich electrochemical aptasensor has been specifically developed using the characteristics of CEA, HRP and concanavalin A (ConA) [135]. The DNA aptamer specifically captured the CEA target to the Au electrode surface. The HRP binds to ConA through sugar–lectin interactions. The DPV signal was measured in the presence of hydroquinone (H_2_Q) and H_2_O_2_.

Table 5 gathers the electrochemical aptasensing assays performances for CEA.

#### 5.4.3. Electrochemical Aptasensors for Exosomes

Exosomes are endosome derived extracellular vesicles of 30–120 nm size ranges. Exosomes serve as mediators of cell–cell communication by the transmission of bioactive molecules such as nucleic acids, proteins, and lipids into receptor cells. Exosomes exist in the tumor medium, indicating a function in facilitating tumorigenesis by regulating angiogenesis, immunity, and metastasis. Circulating exosomes can be used as non-invasive liquid biopsies and biomarkers for the early detection, diagnosis, and treatment of cancer patients [136].

Electrochemical aptasensors for exosome dosages have used an aptamer against CD63 [137]. The CD63 binding aptamer has been used for the capture or detection of exosomes secreted by different cancer cells such as breast (MCF-7) and liver (HepG2) cell lines. Direct electrochemical aptasensing has been developed for exosome detection using an immobilized aptamer specific to capture exosomes. The approach was based on blocking the access of electron transfer from a soluble probe to the electrode surface [138]. In this format, the oriented immobilization of the aptamer on Au with fine spacing through a tetrahedral DNA nanostructure, improves the accessibility of the exosomes to the recognition element and therefore the sensitivity of the test, yielding a much lower LOD than that obtained with an aptamer immobilization using classical SAM.

A displacement test involving immobilization of the aptamer in the form of a duplex, hybridized with one or more strands of antisense DNA has been developed [139,140]. Recognition of the exosomes causes the release of the detected complementary strands. In this case, it is necessary to design the hybridization strands to be complementary to the aptamer in the region directly involved in target recognition and to label the displaced strand with an electroactive marker. As a result, the recognition event produces a decrease in the voltammetric signal of the marker in response to the exosome target.

Exosome recognition can also be transformed into a DNA detection assay by derivatizing exosomes once trapped on the detection layer with a DNA sequence by click chemistry [141]. The ssDNA is then detected by the HCR, using a biotin–hairpin as a probe. The detection of elongated DNA products is achieved by measuring the peroxidase activity of the streptavidin–peroxidase conjugate used as a reporter. Another design based on a recognition event which occurs in solution was also described. The capture aptamer is hybridized with an oligonucleotide which is liberated upon exosome binding. These strands are detected on an ITO electrode using doxorubicin as an electroactive probe by an exonuclease III-assisted recycling assay [142].

The electrochemical detection of HepG2-derived exosomes was based on their enrichment with functionalized anti-CD63 immunobeads, followed by recognition by the DNA chain containing the CD63 aptamer region and a cascade-mediated strand displacement reaction [143]. The on-chip isolation and in situ electrochemical analysis for exosomes steps have been integrated into a microfluidic platform for rapid and simple analysis of exosomes in clinical samples for cancer diagnosis [144].

Table 6 demonstrates performance of electrochemical exosome aptasensing assay.

#### 5.4.4. Electrochemical Aptasensors for Circulating Tumor Cells Biomarkers

Epithelial cell adhesion molecule (EpCAM) is a common biomarker of circulating tumor cells and has an important role in tumor metastasis [145]. It is very difficult to detect EpCAM due to its extremely low concentration in peripheral blood.

An electrochemical aptasensor has been successfully developed for EpCAM detection [146]. The Au electrode has been modified by an aptamer acting as the capture probe for Epcam target, which can then be recognized by a second aptamer acting as a signal reporter. To amplify the signal, a silica/CdSe nanoparticle complex has been devised; silica nanoparticles serve as loading carriers for several CdSe quantum dots capable of binding to a second aptamer via biotin–streptavidin interactions. The EpcCAM molecule can couple to many CdSe quantum dots (CT) to significantly enhancing the SWV signal.

An amperometric aptasensor has been suggested for the detection of EpCAM [147], based on the cooperation of the EpCAM-mediated DNA recycling amplification, the specific recognition of the aptamer EpCAM, and EpCAM binding. The 5′-terminal of the Hairpin 1 probe (Hp1) was modified by the thiol group (5′-SH) and self-organized on the Au electrode surface. After EpCAM addition, the probe A (a 15-sea) is released from the aptamer/probe A complex and then hybridizes with the toehold domain of Hp1. This causes another toehold to be exposed for subsequent hybridization with hairpin probe 2 (Hp2) to move the A probe in the presence of Hp2 labeled with MB. The released probe A is then re-hybridized with another Hp1 to begin the next round of DNA recycling amplification using probe A, resulting in the formation of many strands of MB-labeled DNA on the electrode surface and an amplified current signal.

The nanospheric (CNS)-based aptasensing has been shaped to measure human colon cancer cells DLD-1 through the process of recognition of the MUC-1 aptamer to MUC-1 glyprotein on the cellular membrane that can be used for monitoring the adhesion of DLD-1 cells [148]. The MUC-1 aptamer was bound to the surface of the CNS/GCE activated by carbodiimide chemistry. The MUC-1 aptamer/CNSs/GCE acts as a capture probe for MUC-1. The generated signal was measured by the EIS.

A label-free aptasensor for the detection of Mucin 1 (MUC1), has been suggested [149]. The Au electrode was modified by direct immobilization of thiolated single-stranded anti-MUC1 DNA aptamers which were also labeled at the terminus end with MB. The single strands of DNA are twisted into a hairpin structure which allows direct electron transfer between the MB and Au electrode. The MUC1 bond has changed the conformation of the aptamer, which pushes the redox MB center away from the electrode surface, thus decreasing the detection signal.

A cytosensing technique for the simultaneous multiplex detection of acute myeloid leukemia cells and acute lymphocytic leukemia cells was designed. The design involves the assembly of a multilayer graphene–Au nanoparticle with a dual aptamer function and the use of hybrid electrochemical nanoprobes co-functionalized with an HRP redox marker, cell-targeted nucleic acid aptamers, and the cells being placed between the nanoprobes and the electrode interface [150]. The multifunctional hybrid nanoprobes target specific cells, amplify electrochemical signals, and generate various signals for the multiplex detection of acute myeloid leukemia cells and acute lymphocytic leukemia cells.

Table 7 presents the specifications and the electrochemical performance of aptasensors for circulating tumor cells biomarkers.

## 6. Conclusions and Outlook

Over the last few decades, aptamers have attracted a lot of interest in the biosensor industry, because they are the next generation of target receptors that can replace antibody functions. SELEX is an automated procedure and needs only a few days to evolve some binders. This is much shorter compared to antibody selection, which often requires several months. Aptamers can even differentiate the chirality of a molecule and its secondary structure. Aptamers can choose any types of targets with no restrictions. The antibodies undergo permanent degradation, while aptamers can undergo several cycles of denaturation/regeneration. DNA aptamers are acceptable for the design of reusable aptamer detectors, while RNA aptamers can be single-dimensional. The use of aptamers is not limited to specific areas and can be used as recognition molecules in almost any domain. The main limitation is the degradation of RNAs aptamer by ribonuclease. These problems can be solved by modifying the RNA aptamers. Another limitation is that the microenvironment will affect the structure of the aptamer and the interactions with the ligand target. Moreover, the composition of salts has a significant effect on aptamer configuration. The integration of aptamers into detection platforms such as microfluidics and paper-based analytical devices and lab-on-a-chip (LOC) areas for point-of-care (POC) diagnosis is becoming increasingly popular. Aptamer-based detection systems meet most POC diagnostic requirements.

Selecting a high-affinity aptamer is not a trivial task; there are still many critical biomarkers that do not have suitable aptamers. Therefore, the success of these LOC platforms for an aptamer-based biosensor depends on the discovery of new aptamers that work well for the target analyte. Moreover, the washing step is of crucial importance, because an efficient and rapid target removal procedure is useful for aptasensing in order to obtain continuous and possible real-time detection of the analyte by an automated process. This could improve low-resource health services worldwide.

## Figures and Tables

**Table 1:** Performance and detection methods of different electrochemical cardiac aptasensors.

Cardiac Biomarker	Sensor Specification	Method	Sensing Strategy	Sample Matrices	Detection Rang	Limit of Detection (LOD)	Ref.
Mb	polystyrene microplate	personal glucose meter (PGM) readout	antibody–Mb–aptamer sandwich	serum samples	50 pM–200 nM	50 pM	[71]
cTnI	Au electrode/thiolated apt.	SWV, Fc-SiNPs	label-free	buffer	1−10,000 pM	1.0 pM	[75]
cTnI	AuNPs, Ti Foil/HS-apt.	DPV, [Fe(CN)_6_]^3−/4−^	Label-free	human serum samples	1–1100 pM	0.18 pM	[76]
cTnI	Au electrode/macroporous Au nanostructure/Apt.	DPV, [Fe(CN)_6_]^3−/4−^	label-free	spiked human serum samples	0.05–5.0 ng mL^−1^	23 pg mL^−1^	[77]
TnI	array of Au nanodumbbells/76-mer TnI aptamer	DPV [Fe(CN)_6_]^3−/4−^	label-free	blood serum samples	0.05–500 ng mL^−1^	8.0 pg mL^−1^	[78]
cTnI	SPCE/AuNPs/electropolymerized conductive polymer/Apt.	amperometry, hydrazine, H_2_O_2_	apt. pair-based sandwich type	serum	1–100 pM (0.024–2.4 ng/mL)	1.0 pM (24 pg/mL)	[79]
cTnI	GC/N-prGO/COOH/PEG-apt.	DPV	Label-free	serum, saliva	0.001–100 ng mL^−1^	0.88 pg mL^−1^	[80]
cTnI	Apt.–MoS2 nanosheets	EIS, [Fe(CN)_6_]^3−/4−^	label-free	human blood samples	10 pM to 1.0 μM	0.95 pM	[81]
	Au@SiO2@Au/aptamer-				10 pM–10.0 μM	1.23 pM	
cTnI	DNA nanotetrahedron (NTH)/dual-apt. (dAPT), magnetic metal organic frameworks (mMOFs), Fe_3_O_4_@UiO-66, Au@Pt NPs	DPV hydroquinone, H_2_O_2_	sandwich-structure	human serum	0.01–100 ng·mL^−1^	5.7 pg mL^−1^	[82]
cTnI	Au electrode/apt. probe 2 (P2)	SWV MB	sandwich-structure	human serum, urine saliva	0.5–100 ngmL^−1^	0.04 ngmL^−1^	[83]
Mb	apt./rGO/CNT nanohybrid electrodes	CV direct electron transfer Mb	label-free	buffer	1 ng/mL–4 µg/mL	~0.34 ng/mL	[84]
Mb	Y-shape structure of dual-aptamer (DApt)-	DPV [Fe(CN)_6_]^3−/4−^	label-free	human blood serum	100 pM–40 nM	27 pM	[85]
Mb	apt.–black phosphorus nanostructured electrode	CV	direct electron transfer Mb	serum samples	1 pg mL^−1^–16 µg mL^−1^	0.524 pg mL^−1^	[86]
Mb	meso-tetra (4-carboxyphenyl) porphyrin-AuNPs(TCPP–Gr/AuNPs)/apt. Mb	DPV	electron transfer by Mb	human serum samples	2.0 × 10^−11^–7.7 × 10^−7^ M	6.7 × 10^−12^ M	[87]
Lys	Au electrode/Lys apt.	SWV Fc, [Ru(NH3)_6_]^3+^	signal on/off, label-free	human serum	1.0 × 10^−11^–1.0 × 10^−7^ M	0.8 pM	[88]
Mb	GCE, PtSnNP CNTs/Mb-apt.	Amperometry, [Fe(CN)_6_]^3−/4-^	conformational change	Spiked human serum samples	0.01–1 and 10 nM–200 nM	2.2 ± 0.1 pM	[89]
Mb	Apt/AuNPs/BNNSs/FTO	DPV [Fe(CN)_6_]^3−/4−^	label free	human serum Hb, GOx, Ins., SOx	0.1–100 µg/mL	34.6 ng/mL	[90]
cTnI Mb	HsGDY@NDs/apt.strand	EIS [Fe(CN)_6_]^3−/4−^	label-free	human serum	cTnI 0.00001–100 ng mL^−1^ Mb 0.01–1000 pg mL^−1^	cTnI 9.04 fg mL^−1^ Mb 9.04 fg mL^−1^	[91]

**Table 2:** Specifications of electrochemical aptasensors for Alzheimer’s disease (AD) biomarkers.

AD Biomarkers	Sensor Specification	Method	Sensing Strategy	Sample Matrices	Detection Rang	Limit of Detection (LOD)	Ref.
tau 381	apt./carboxyl Gr/th./AuNPs GCE	DPV Th. redox probe	label-free	10 patients’ serum samples	1.0–100 pM	0.70 pM	[92]
tau 381	tau antibody (anti-tau)/apt.specific to tau-381	EIS, CV, DPV AuNPs signal amplification	Apt.-antibody sandwich	AD patients serum	0.5–100 pM	0.42 pM	[93]
AβO	*β* oligomers antibodies/nanocomposite AuNPs/apt.	DPV Th. redox probe	antibody-apt. sandwich	artificial cerebrospinal fluid (CSF)	0.5–30 nM	100 pM	[94]
AβO	SAM ssDNA apt./Au electrode	EIS [Fe(CN)_6_]^3−/4−^	label-free	artificial CSF	0.1–500 nM	0.03 nM	[96]
AβO	AuNFs/GCE/HS-apt.	DPV MOFs signalprobe	sandwich	artificial CSF	1 nM–2 µM	0.45 nM	[97]
α-synuclein	Apt./SAM	DPV MB	label-free	human serum samples	60 pM–150 nM	10 pM	[98]
AβO ATP	nanostructured multielectrode arrays (MEAs); dual-apt.	Amperometry Fc	signal-on/signal-off	artificial CSF	1 pM–200 nM 0.01 nM–1000 nM	0.3 pM 0.002 nM	[99]

**Table 3:** Specifications of electrochemical aptasensors for infectious disease biomarkers.

Infectious Disease Biomarkers	Sensor Specification	Method	Sensing Strategy	Sample Matrices	Detection Range	Limit of Detection (LOD)	Ref.
IFN-γ	SAM DNA hairpin IFN-γ-apt./MB/Au electrode	SWV	conformational changes	serum proteins	0.06–10 nM	0.06 nM	[101]
bovine interferon gamma (BoIFN-γ)	SAM IFN-γ-binding apt. MB	SWV	conformational change	pristine buffer, blood	1–230 ng/mL	pristine buffer 0.1 nM blood 0.9 nM	[102]
IFN-γ	IFN-γ apt./Gr surface	conductance	change in charge distribution in electrolyte	BSA, papain	0 nM–5 μM	83 pM	[103]
IFN-γ	SiNWs/Au/thiolated apt.	SWV MB	conformational change, Switch off	blood serum	nanowire 3 pg/mL for 5000 cells—planar 90 pg/mL for 30,000 cells	0.14 ng/mL, 0.8 ng/mL	[104]
IFN-γ TNF- α	micropatterned Au electrodes arrays/cytokine-binding apt./microfluidic device	SWV	conformational change	immune cells	0.0–100 ng/mL (5.8 nM)	10 ng/mL (0.58 nM)	[105]
C. perfringens pathogen	capture probe, streptavidin (SA), hemin/G-quadruplex/Fe_3_O_4_ nanocomposites	DPV	sandwich	Real biological samples	10^−12^–10^−6^ M	10^−12^ M	[106]
NS-1 protein	Pencil electrode (PE)/(BE)/PEI/AuNPs/apt.	CV [Fe (CN)6]^−3/−4^	label-free, structural switching	spiked human serum sample	3–160 ng mL^−1^	0.3 ng mL^−1^	[107]

**Table 4:** Specifications and electrochemical performance of CTCs aptasensors

CTCs	Sensor Specification	Method	Sensing Strategy	Sample Matrices/ Interferents	Detection Range	Limit of Detection (LOD)	Ref.
MEAR cancer cells	dual-apt. TLS1c, TLS11/amide bond/GCE	DPV [Fe(CN)_6_]^3−/4−^	label-free	blood cells	1−14 MEAR cells	single MEAR cell/10^9^ whole blood cells	[110]
CTCs	EpCAM apt./MCH/Au electrode	EIS [Fe(CN)_6_]^3−/4−^	label-free	real blood sample	30–1 × 10^6^ cells L^−1^	10 cells mL^−1^	[111]
acute leukemia CCRF-CEM	Apt./array nanochannel–ionchannel hybrid	LSV	label-free	k562 cells, Ramos cells	1 × 10^2^–2 × 10^6^ cells mL^−1^	100 cells mL^−1^	[112]
MCF-7 circulating tumor cells	MUC-1 apt./rGO/AuNPs composites	DPV CuO NPs, H_2_O_2_	label-free	serum sample	50–7 × 10^3^ cells mL^−1^	27 cells mL^−1^	[64]
Ramos, CCRF-CEM cells	Apt.–AuNPs Array/Magnetic Gr Nanosheets	SWASV multiplexed detection	label-free	human whole blood	5–500 cells mL^−1^	Ramos 4 cells mL^−1^ CCRF-CEM cells 3 cells mL^−1^	[62]
CTCs K562 cell	AuNPs/Au electrode, MB–apt.	Alternating current voltammetry (ACV) MB	signal-off	U937, Jurkat cells	1 × 10^2^–1 × 10^6^ cells mL^−1^	23 cells mL^−1^	[114]
Breast cancer cell MCF-7	Fe_3_O_4_ magnetic nanospheres (MNs)/EpCAM antibody	SWV molybdophosphate (PMo12O40) redox label	antibody–aptamer sandwich type	whole blood samples	3–10,000 cell mL^−1^	1 cell mL^−1^	[115]
MCF-7 cells	Membrane protein MUC1-targeting apt.	dual signal, (Ce@IrNRs) enzyme-free DNA walker	enzyme-free DNA walker	whole blood samples	2–2 × 10^6^ cells mL^−1^	1 cell mL^−1^	[116]
CTCs MCF-7	Apt. magnetic beads MBs	DPV ferroceneboronic acid (FcBA)	label-free strategy	blood sample	50^−2^ × 10^4^ cells	50 cells	[117]

**Table 5:** Specifications and electrochemical performance of aptasensors for carcinoembryonic antigen (CEA).

Sensor Specification	Method	Sensing Strategy	Sample Matrices	Detection Range	Limit of Detection (LOD)	Ref.
apt1/Au	DPV Fc	sandwich-type	human serum spiked	1–200 ng/mL	0.5 ng/mL	[119]
dendritic Pt@AuNWs nanocarriers/thiol- CEA apt. 2	DPV Tb, Pt@AuNWs H_2_O_2_	sandwich-type	human serum sample	0.001–80 ng mL^−1^	0.31 pg mL^−1^	[120]
Au/DNA1/MCH/DNA2/DNA3-MB electrode	SWV MB	label-free	tumor necrosis factor-α (TNF-α), thrombin	10–100 nM (MUC1) 30–300 ng mL^−1^ (CEA)	0.13 nM (MUC1) 2.75 ng mL^−1^ (CEA)	[121]
aptamer/hemin and graphene nanosheets (AuNPs-HGNs)/GCE	DPV electron transfer of hemin	label-free	human serum albumin HSA, thrombin (TB), lysozyme (LYS), insulin (INS)	0.0001–10 ng mL^−1^	40 fg mL^−1^	[121]
MCH/ITO/Au film CEA apt./anode pole bipolar electrode (BPE)	EIS PB	structural change	human serum; AFP, BSA, thrombin	1–10 ng mL^−1^	-	[122]
hairpin oligonucleotide apt./Au nanorods/Gr-streptavidin nanomatrix/GCE	DPV HRP, H_2_O_2,_ o-Phenylenediamine	switch-on	8 human serum samples, lung diseases patients	5 pg mL^−1^–50 ng mL^−1^	1.5 pg mL^−1^	[123]
PtPdNWs/CEA apt.	EIS oxidation DAB	label-free	human serum samples	0.1–0.5 ng mL^−1^ and 1–40 ng mL^−1^	0.030 pg mL^−1^	[124]
BSA/aptamer/AuNPs /MoSe2-Gr/GCE	DPV [Fe(CN)6]^3−/4−^	structural change switch-on	HAS, IgE, AFP, Thrombin, LDL	0.1 pg mL^−1^–100 ng mL^−1^	0.03 pg mL^−1^	[125]
amine–Apt./AuNPs/Mesoporous Silica Films/GCE	EIS [Fe(CN)6]^3−/4−^	structural change	patient, healthy human serum samples	1.0 × 10^−3^−100.0 ng mL^−1^	9.8 × 10^−4^ ng mL^−1^	[126]
509-MOFs@Apt. strand	EIS [Fe(CN)_6_]^3-/4-^	conformational changes, label-free	human serum sample	0.001–0.50 ng mL^−1^	CEA 0.40 pg mL^−1^, thrombin 0.37 pg mL^−1^, Kanamycin 0.21 pg mL^−1^	[127]
GCE/Au nanorods/Antibody	redox-active PMo_12_O_40_	sandwich-type	human serum samples	0.1 pg mL^−1^–10 ng mL^−1^	0.05 pg mL^−1^	[128]
MCH/apt./(ISP)/AuNPs/GCE	SWV MB Fc	DNA structural switching	HAS, HIgG	1–1 μg mL^−1^ (CEA), 5 nM–1 μM (MUC1)	0.517 ng mL^−1^ (CEA), 1.0^6^ nM (MUC1)	[129]
Zr-MOF AgNCs@Apt@UiO-66, AgNCs@CEA-apt.	EIS, DPV, SPR	label-free	human serum samples	0.01−10 ng mL^−1^ (EIS, DPV) 1.0−250 ng mL^−1^ (SPR)	8.88 (EIS) 4.93 (DPV) pg mL^−1^ 0.3 ng mL^−1^ (SPR)	[130]
GCE/AuNP–apt.	SWV redox-active PMo_12_O_40_ rolling circle amplification (RCA)	sandwich-type	human serum samples	0.5 pg mL^−1^–1 ng mL^−1^	0.1 pg mL^−1^	[131]
GCE/apt./CuMOF/Pt	EIS 3DAB, insoluble precipitates (IPs)	sandwich-type	human serum samples	0.05 pg mL^−1^–20 ng mL^−1^	0.023 pg mL^−1^	[132]
GQDs-IL-NF/glassy carbon electrode/single-stranded DNA	DPV MB, Pb^2+^ DNAzyme- signal amplification	label- free	serum samples; CEA, PSA, BSA, MUC1	0.5 fg mL^−1^–0.5 ng mL^−1^	0.34 fg mL^−1^	[133]
ADH/hemin/G-quadruplex (hGQ-peptide)/apt./PdNPs/GCE	DPV Tb, ADH	sandwich-type	serum sample; AFP, BSA, Hb	0.0001–100 ng mL^−1^	20 fg mL^−1^	[134]
MCH–Apt./Au	DPV HRP label	label-free, sandwich-type	human blood serum samples; BSA, HSA, γ-glubulin, AFP, CRP	5–40 ng mL^−1^	3.4 ng mL^−1^	[135]

**Table 6:** Specifications and electrochemical performance of aptasensors for exosomes.

Exosomes	Sensor Specification	Method	Sensing Strategy	Sample Matrices	Detection Range	Limit of Detection (LOD)	Ref.
MCF-7 exosomes	Apt./2D Ti3C2 MXenes nanosheets	Electrogenerated chemiluminescence (ECL)	sandwich-type	serum	5 × 10^2^–5 × 10^6^ particles μL^−1^	125 particles μL^−1^	[137]
hepatocellular exosomes	Apt.-nanotetrahedron (NTH) SAM/Au electrode	SWV [Fe(CN)_6_]^−3/−4^	sandwich-type	membrane proteins	10^5^–10^12^ exosomes/mL	1 × 10^4^/mL	[138]
exosomes	exosome transmembrane protein CD63 apt./Au electrode array, microfluidic system	SWV MB redox label	switch-off	fetal bovine serum	1 × 10^8^ particles/mL	1 × 10^6^ particles/mL	[139]
tumor exosomes LNCaP cells	DNAs/Au electrode	DPV Ru(NH_3_)_6_^3−^ signal reporter	“turn-off” signal	MCF-7 cell, HeLa cell-derived exosomes	1000–120,000 particles/µL	70 particles/µL	[140]
Exosomes	CD63 apt./GCE/Cu (I)-catalyzed click chemistry	DPV HRP, o-phenylene-diamine (OPD), H_2_O_2_	label-free	human serum	1.12 × 10^2^ to 1.12 × 10^8^ particles/μL	96 particles/μL	[141]
cancer-exosome	ITO electrode	DPV DOX redox-active indicator	label-free “signal-on” strategy	biological, clinical samples	3.4 × 10^4^–3.4 × 10^8^ particles per mL	1.2 × 10^4^ particles per mL	[142]
HepG2-exosomes	Au electrode/apt.	SWV [Fe(CN)_6_]^−3/−4^	displacement reaction	serum samples	1 × 10^5^–5 × 10^7^ particles/mL	1.72 × 10^4^ particles/mL	[143]
CD63 positive exosomes	ITO electrode microfluidic on-chip platform (ExoPCD-chip)	DPV [Fe(CN)_6_]^−3/−4^, th. enhanced cathodic current	label-free	human serum and liver cancer patients, healthy controls	7.61 × 10^4^ particles/mL to 7.61 × 10^8^ particles/mL	4.39 × 10^3^ particles/mL	[144]

**Table 7:** Specifications and electrochemical performance of aptasensors for circulating tumor cells biomarkers.

Circulating Tumor Cells Biomarkers	Sensor Specification	Method	Sensing Strategy	Sample Matrices/Tested Interferents	Detection Range	Limit of Detection (LOD)	Ref.
EpCAM	Au electrode/2-mercaptoethanol/apt.	stripping SWV CdSe NPs signal	sandwich-type	added CK-19, thrombin	10 aM to 100 pM	10 aM	[146]
EpCAM	EpCAM apt. Hairpin probe 1 (Hp1) 5′-SH/SAM/MCH/Au electrode	SWV MB	switch-on	spiked human serum, urine, saliva	0.1–20 ng mL^−1^	20 pg mL^−1^	[147]
human colon cancer DLD-1 cells	MUC-1 apt./CNSs	EIS, CV [Fe(CN)_6_]^−3/−4^	label-free	human astrocyte (HA) cells, DLD-1 cells	1.25 × 10^2^–1.25 × 10^6^ cells per mL	40 cells per mL	[148]
epithelial tumor marker mucin 1 (MUC1)	MB-hairpin DNA apt./Au electrode	SWV	conformational change switch-off	added lysozyme, cytochrome c, and BSA	up to 1.5 M	50 nM	[149]
human leukemia cell line (HL-60) lymphocytic leukemia cells (CEM cells)	dual apt./multilayered graphene–AuNPs electrode	DPV HRP redox tags	sandwich-type	Hela, K562, normal red blood cells	5 × 10^2^–1 × 10^7^ cells per mL	350 cells per mL	[150]
